# 

**Published:** 1890-07

**Authors:** 


CONTENTS:

PAGE

ORIGINAL ARTICLES:

Texas Fever (Southern Cattle Fever, Etc.). By Paul Paquin . . . . . . . 367
Tumor of Spinal Cord. By John M. Parker, D.V.S. . . . . . . . . . . . . 384
Mad Itch. By S. Stewart, M.D., D.V.M. . . . . . . . . . . . . . . . . . 386
Age of the Horse, Ox, Dog, and other Domesticated Animals. By R. S. Huidekoper, M.D., Veterinarian . . . . . . . . . . . . . . . . . . . . 390
Tympanitis Cured with Carbolic Acid. By Claude D. Morris, V.S. . . . . 398
Resistance to Hydrocyanic Acid Poisoning. . . . . . . . . . . . . . . . 398
A Legal Decision. . . . . . . . . . . . . . . . . . . . . . . . . . . . 399
A Correction . . . . . . . . . . . . . . . . . . . . . . . . . . . . . . 400
Notice . . . . . . . . . . . . . . . . . . . . . . . . . . . . . . . . . 400

SOCIETY PROCEEDINGS:

The Kansas Veterinary Medical Society . . . . . . . . . . . . . . . . . 401
Keystone Veterinary Medical Association. . . . . . . . . . . . . . . . 402
Massachusetts Veterinary Association. . . . . . . . . . . . . . . . . 403
Joint Meeting of the Indiana and Illinois Veterinary Medical Associations. 406
Long Island Veterinary Society.. . . . . . . . . . . . . . . . . . . . 411

VETERINARY COLLEGE NOTES:

Veterinary Department, Harvard University.. . . . . . . . . . . . . . . 412

BOOKS AND PAMPHLETS RECEIVED. . . . . . . . . . . . . . . . . . . . . 413

